# Kinematic Analysis of 360° Turning in Stroke Survivors Using Wearable Motion Sensors

**DOI:** 10.3390/s22010385

**Published:** 2022-01-05

**Authors:** Masoud Abdollahi, Pranav Madhav Kuber, Michael Shiraishi, Rahul Soangra, Ehsan Rashedi

**Affiliations:** 1 Department of Industrial and Systems Engineering, Rochester Institute of Technology, Rochester, NY 14623, USA; ma8489@rit.edu (M.A.); pmk2015@rit.edu (P.M.K.); 2 Department of Physical Therapy, Crean College of Health and Behavioral Sciences, Chapman University, Orange, CA 92866, USA; shiraishi@chapman.edu (M.S.); soangra@chapman.edu (R.S.); 3 Fowler School of Engineering, Chapman University, Orange, CA 92866, USA

**Keywords:** neurological disorder, motion analysis, inertial measurement unit, stroke, turning

## Abstract

Background: A stroke often bequeaths surviving patients with impaired neuromusculoskeletal systems subjecting them to increased risk of injury (e.g., due to falls) even during activities of daily living. The risk of injuries to such individuals can be related to alterations in their movement. Using inertial sensors to record the digital biomarkers during turning could reveal the relevant turning alterations. Objectives: In this study, movement alterations in stroke survivors (SS) were studied and compared to healthy individuals (HI) in the entire turning task due to its requirement of synergistic application of multiple bodily systems. Methods: The motion of 28 participants (14 SS, 14 HI) during turning was captured using a set of four Inertial Measurement Units, placed on their sternum, sacrum, and both shanks. The motion signals were segmented using the temporal and spatial segmentation of the data from the leading and trailing shanks. Several kinematic parameters, including the range of motion and angular velocity of the four body segments, turning time, the number of cycles involved in the turning task, and portion of the stance phase while turning, were extracted for each participant. Results: The results of temporal processing of the data and comparison between the SS and HI showed that SS had more cycles involved in turning, turn duration, stance phase, range of motion in flexion–extension, and lateral bending for sternum and sacrum (*p*-value < 0.035). However, HI exhibited larger angular velocity in flexion–extension for all four segments. The results of the spatial processing, in agreement with the prior method, showed no difference between the range of motion in flexion–extension of both shanks (*p*-value > 0.08). However, it revealed that the angular velocity of the shanks of leading and trailing legs in the direction of turn was more extensive in the HI (*p*-value < 0.01). Conclusions: The changes in upper/lower body segments of SS could be adequately identified and quantified by IMU sensors. The identified kinematic changes in SS, such as the lower flexion–extension angular velocity of the four body segments and larger lateral bending range of motion in sternum and sacrum compared to HI in turning, could be due to the lack of proper core stability and effect of turning on vestibular system of the participants. This research could facilitate the development of a targeted and efficient rehabilitation program focusing on the affected aspects of turning movement for the stroke community.

## 1. Introduction

Stroke, an emergency condition caused by restriction/interruption of blood supply to the brain, remains among the most common neurological disorders and one of the leading causes of disability. Surviving a stroke, by itself a feat, often imposes considerable debilitations on the patient. Besides post-stroke weakness, stroke survivors (SS) often experience balance, coordination, and movement restriction issues, wherein rehabilitation may be helpful. Rehabilitation programs aim for recovery by developing and providing activities for patients to re-learn lost functions. These activities may include physical, technology-assisted, or cognitive and are determined based on affected regions of the body [1]. With a prevalence of ~7 million SS in the U.S. alone, high financial demand of ~75 billion USD (2013) is imposed on the healthcare system [2], with average yearly rehabilitation costs per patient amounting ~11,000 USD [3]. Meanwhile, literature reports a wide variability in rehabilitation programs stemming from responsiveness to treatment, clinical factors (age, gender, underlying disorders, etc.), and injury (location, volume, type, etc.). Moreover, variation in the amount and severity of such factors can be among the reasons for the variation in success rates, costs incurred, and recovery duration [4]. To provide effective rehabilitation, successful identification of problem areas in the body is critical [5] and can be done by studying the movement of stroke patients.

Examining movement aberrations in stroke patients helps identify the issues and could also help prevent future injuries. Movement limitation in patients can lead to reduced stability (increased fall/injury risk), which could be fatal [6,7]. Thus, several scales, classified according to body function/structure, activity, and participation (social roles), have been developed to test several deficiencies in patients [8]. Among the many scales to measure physiological, neurological, cognitive, psychological, and perception parameters; those that measure body capabilities include Fugl-Meyer (Motor, balance, range of motion, joint pain), Orpington Prognostic Scale (Motor deficit in arm, balance), and Stroke Rehabilitation Assessment of Movement (upper/lower extremity mobility) [8]. These scales measure body function. However, scales like Action Research Arm Test (grasp, grip, pinch, etc.), Box and Block Test (manual dexterity), Chedoke-McMaster Stroke Assessment Scale (postural control, walking), six-minute walk (6MW) test (walking) and Timed “Up and Go” (TUG) Test (walking and turning) measure capabilities in performing movement related activities. Besides TUG and 6MW, Figure of Eight Test (FET) is used to measure advanced walking performance by introducing curved walking, a challenge to stroke patients due to shift in center of mass on inner leg [9]. However, the overall time used as an output measure in these tests may not highlight specific issues behind the reduced performance [10]. Thus, recent studies [11,12] propose utilizing motion analysis to assess better, compare, and interpret patients’ movements in a much higher resolution.

Although motion analysis can help provide a deeper understanding of underlying mechanisms behind the reduced performance, only a few studies have used advanced motion capture systems for stroke patients. For instance, analyzing locomotor trajectories in TUG showed that phase preceding turning sub-task distinguished patients with risk of fall and proposed that rehabilitation focus should be on this phase [13]. Meanwhile, one study [14] showed that the length of the walkway affected distance in 6MW instead of turning direction. Bonnyaud et al. [10], identified specific gait parameters related to TUG performance using an optoelectronic motion capture system and proposed targeted rehabilitation. A common observation in the literature shows that almost all studies consider pre-defined tests, such as TUG, for analyzing motion. However, a standard TUG considers a combination of turning and walking, which may not always be the case. Several activities of daily living (ADL) require on-spot turning, which rely on balance and axial control. A recent study [15] assessed turning by measuring time, steps, strategy, and balance in a 180-degree on-spot turn and found majority of stroke patients experienced turning difficulty. This shows that there is a need for a more robust analysis of specific sub-tasks of turning.

Recent advances in sensor technologies have paved the way for more accessible and portable motion capture systems, which use Inertial Measurement Unit (IMU) and allow for outside-of-lab assessment. The goal of this article would be to utilize these benefits to identify the changes in movement among stroke patients during full 360-degree turning. To this end, we will extract several spatio-temporal measures from four motion sensors placed on the sternum, sacrum, and shanks. These measures were compared between stroke survivors and their healthy counterparts. Through the comparisons, the significantly different parameters among a pre-decided set of kinematic measures were identified. Improvement in mobility in SS has been observed up to the first three months after stroke [16]. The parameters in this study could potentially provide efficiently targeted rehabilitation to stroke survivors, which may be more effective with potentially higher success rates. Moreover, the work presented in this article could be used as a performance monitoring tool and may be more beneficial in obtaining better insights into the condition of stroke patients.

## 2. Materials and Methods

The framework of the study consisted of a controlled laboratory study using the 360-degree turning performed by SS and healthy individuals (HI), followed by data analysis techniques for extracting/determining kinematic output measures, as described in detail in the following sections.

### 2.1. Participants

The study involved the recruitment of 28 human subjects (14 SS, 14 HI) with demographic information, as shown in Table 1. The sample size was determined based on a power of 0.8 with an effect size of 0.92, as determined through power analysis using the G-Power software [17]. Prior to conducting the study, written consent was obtained from all participants and was approved by the Institutional Review Board of Chapman University. Participants in the study were selected based on an inclusion criteria that (a) they should be capable of performing non-assisted straight-line walking up to/more than a distance of 10 m, (b) possess a lack of cognitive impairment measured by Mini-Mental State Examination (MMSE > 24), (c) no co-morbidity that could limit the performance of sit-to-stand was present, and (d) have had suffered stroke no less than 6 months till the experimentation date. Among the participants in the SS group, stroke affected the right side of seven participants and the remaining seven had the left side of their body affected. Two licensed physical therapists were present during the study on each side of the participant to avoid any potential falls during the trials.

### 2.2. Apparatus

**Figure 1:**
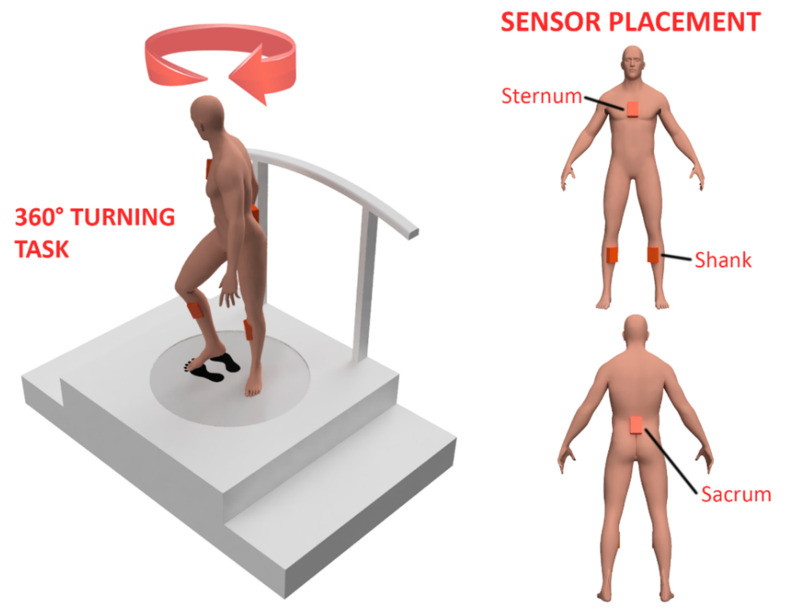
An illustration depicting (**left**) a participant performing a 360° turning task along with (**right**) the locations for the placement of sensors showing the sternum, sacrum, and shanks of a mannequin representing a participant.

A portable IMU-based system (Xsens, Enschede, The Netherlands) in a four-sensor configuration (Sternum, Sacrum, and each of shanks) was used for the measuring participants’ movement at a sampling rate of 60 Hz while they performed the 360-degree turning task (Figure 1). A software developed by the same firm was used for obtaining data related to the movement of the respective regions of participants’ body. Intending to include the minimum number of sensors to ensure accessibility for stroke patients, we chose the four-sensor configuration among the provided choices of full-body, upper-body, and four-sensor configuration. This configuration provided motion data not only from the upper and lower body but also from the two sides of the lower body. Obtaining movement data from both sides can allow us to compare the movement discrepancies between the leading and trailing leg during turning. Setup for experimentation included an open area without obstacles and with markers on the floor (representing footprints) for the initial and end positions of the participant while performing on-spot turning. Meanwhile, a target was placed on virtual screen when facing the initial and end positions of the participant during the turning task. In order to ensure participants’ safety, a harness attached to a rail on the ceiling was used while they performed the tasks.

### 2.3. Experimental Task

Prior to experimentation, the motion capture system was calibrated according to their respective anthropometric measurements for reliable data collection using the N-Pose pre-defined setup in the software. Participants were then instructed to stand on the footprint marker placed on the floor and look straight. Specifically, they were told to avoid looking at the floor as they performed the task. Each participant performed an on-spot 360-degree turning at their comfortable pace with the end-position indicated to the participant with the help of a virtual target.

### 2.4. Data Analysis

The collected data from the motion capture system was exported into MATLAB^®^ (Mathworks, Inc., Natick, MA, USA) using a custom-made code provided by Xsens. The exported files included raw kinematic data consisting of linear acceleration, angular velocity, and orientation in a 3D space for each of the four sensors located on the sternum, sacrum, and shanks. After extracting the data, a custom program was written to analyze and extract critical parameters from data.

#### 2.4.1. Parameter Selection

The IMU sensors collected linear acceleration, angular velocity, and orientation from the four segments. However, turning motion involves mostly on-spot movement and a lack of sufficient translational movement, which may reduce the relevance of linear parameters; thus, only angular parameters were considered in this study. The initial list of parameters included the angles, angular velocity, and angular acceleration along all three axes, number of cycles in each turning task, duration of each turning cycle, double support, and single support phases of foot-floor contact. Moreover, the relative angular velocity and range of motion between the two shanks and sacrum-sternum were also considered. Table 2 shows the selected parameters per segment that were considered for the next stage of motion analysis.

#### 2.4.2. Segmentation and Feature Extraction

The shank sensors were considered for segmenting the data due to having wider maximal-minimal deviations, which increases segmentation accuracy. Among the two available sensors placed on participants’ leading and trailing legs, the sensor placed on the leading leg was selected for temporal processing. The sensor placed on the trailing leg was considered for spatial segmentation. This stemmed from the fact that a stroke survivor would select the unaffected leg as the leading leg while selecting the direction of turning. Thus, being in the affected region, the trailing leg did not show a clear pattern that could be used for effective temporal segmentation. However, in contrast to temporal segmentation, spatial segmentation offers independence from patterns in the movement and could be implemented to analyze the trailing leg’s movement.

**Figure 2:**
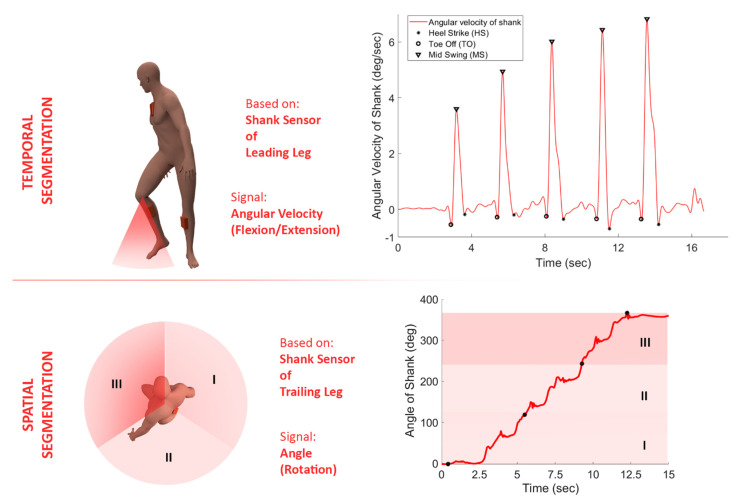
An illustration depicting the method, sensor, and type of signal used for spatial and temporal segmentation of the obtained motion data.

Temporal processing was conducted on the leading leg by using the angular velocity signal in the flexion–extension direction (Figure 2). The exported data from the Xsens software (MVN Analyze^®^, Xsens, Enschede, The Netherlands) was filtered using a 4th order low-pass Butterworth filter with a cut-off frequency of 5 Hz. Based on preliminary observation, each swing of the leading leg during the turning task would appear as a bell shape, with angular velocity signal increasing to a maximal point and then decreasing. Since the turning task would include multiple swing and stance cycles, points of repetition of this pattern can be used for event detection of mid-swing, toe-off, and heel-strike points. Figure 2 (top-right) shows the segmented signal for one of the participants.

The orientation of the shank sensor placed was used for spatial processing of the movement of the trailing leg. Generally, all the participants covered ~360° rotation to complete the task. To segment signals, we could partition this interval by different steps. Since the number of partitions on the outcome of the analysis was unknown, the 360° movement was divided into 2, 3, 4, …, 7 different partitions. Figure 2 (bottom-left) shows the 360° turning movement is divided into 3 partitions. To calculate other parameters, we would need the time indices of each partition. To determine the time indices of the partitions, a custom program was developed to divide the rotation angle of the sensor on the shank of the trailing leg into the different beforementioned partitions and extract the time indices for the start/end of each partition. The subsequent figure on the bottom-right (Figure 2) shows the signal being segmented according to the shank angle.

After performing the segmentation on both of the sensors on the shank, the earlier mentioned parameters were calculated by averaging the corresponding type of signal extracted from the software (MVN Analyze^®^) for each segment/partition. Finally, mean and standard deviation were calculated for each group and compared using an unpaired two-tailed *t*-test in MATLAB^®^. The significance level in all statistical analyses was considered to be 0.05.

## 3. Results

The kinematic measures were calculated in both segmentation methods, including temporal and spatial. The parameters were compared for the SS and HI. The kinematic parameters extracted from temporal segmentation of the angular velocity signal (e.g., duration of the turn and range of motion of sacrum in flexion/extension direction) are presented in Table 3. Furthermore, the kinematic parameters calculated based on the spatial segmentation (e.g., mean angular velocity of shanks in flexion/extension direction) have been displayed in Table 4. Except for the range of motion in flexion/extension for the leading leg, significant differences were observed in all parameters between SS and HI control groups for the temporal processing of motion signals. The SS group showed higher values of the number of cycles, duration of the turn, portion of stance phase in each cycle, and range of motion for sternum and sacrum in lateral bending and flexion–extension.

Meanwhile, significant differences were not obtained for spatial processing for the angular velocity in flexion–extension direction for the leading and trailing leg and angular velocity of the sternum in rotation. Except these, significant differences were obtained in either of the two or four partitions for the spatial segmentation processing. Table 4 shows the comparison between the angular velocities of each segment, while the range of motion comparison is displayed in Table 5. The relative measures of range of motion and angular velocity have been depicted in Table 6, which shows the comparison between the two groups for the two and four partitions. The results show that flexion–extension angular velocity of the four body segments in two-partition segmentation was significantly larger in HI (*p*-value < 0.026). However, by adding to the number of partitions, some of the partitions showed non-significant changes, which could help in-depth analysis of the movement. Moreover, the rotational angular velocity of the sternum and sacrum were similar in the two-partition segmentation. However, this parameter was larger in HI shanks (*p*-value < 0.005). Furthermore, the range of flexion–extension motion in sternum and sacrum was larger in SS in two-partition segmentation; however, it was similar for the shanks. Finally, according to the results of the two-partition segmentation, the range of motion for lateral bending of the sternum was more extensive in SS (*p*-value < 0.02). However, it was not significantly different for the sacrum body segment.

## 4. Discussion

Stroke impacts patients’ daily routine by hindering their movement capabilities, which may lead to falls and/or severe injuries. Movement analysis has been considered as an effective method of identifying the affected body regions, level of recovery and developing rehabilitation programs/interventions in the stroke community. Prior studies on movement analysis have been largely focused on overall parameters like velocity and time to complete the task [18]. While a few studies have used a motion capture system to detect relative movements of individual segments, these studies have considered walking, or a combination of walking and turning [10,13,19]. In addition to walking, turning is another most-common activity of daily living. However, it was barely considered in the literature, with only a few studies [15,20], to conduct an in-depth analysis and identify the kinematic changes in SS. Although Soangra et al. [21] conducted a study on the 360-degree turn using camera-based motion capture system, there was an opportunity to make the assessment more accurate and accessible by using IMU sensors and adding to the pool of measures to be analyzed and compared between the SS and HI groups. To pave a pathway towards effective rehabilitation by targeting affected regions, we have investigated the movement during a complete turning task in this study. An experiment was conducted by placing IMU sensors on the sternum, sacrum, and shanks of the participants; and recording their movement as they performed a turning task. The analysis procedure included segmentation of both the leading and trailing legs of each participant. While the data obtained from the leading leg consisted of temporal segmentation, the trailing leg was segmented using spatial processing. After segmenting, a set of parameters were analyzed, which represented a simplified version of the movement characteristics in the three-dimensional environment, which can be easily understood. This study shows that using wearable sensors movement discrepancies in stroke survivors can be successfully identified and differentiated with healthy individuals.

The results of temporal processing of the data depicted that SS group was slower, as expected, in turn. These results are in agreement with literature confirming the slower motion of SS in walking [21,22,23]. Specifically, the **number of cycles** and **turn duration** were 67% (*p* =0.001) and 106% (*p* = 0.004), respectively larger in the SS group. These higher values could be due to difficulties experienced by SS in turning as compared to HI. To understand these effects in further depth, other parameters can be evaluated and compared. For example, the portion of **stance phase** in turning consisted of ~53% among SS, as opposed to ~39% in HI. Lack of balance when experienced during movement, an individual will tend to place both feet firmly (similar to a stance phase) on the ground. Literature shows that the vestibular system, which is known to provide a sense of balance to an individual, when affected, can result in slower movement [24]. A previous study imposed cognitive loadings on SS as they turned and walked, resulting in slowed movement [19]. The SS performed the activity by having both feet on the ground more frequently than HI group, which could have resulted in the higher **duration of turn and number of cycles**. This could mean that the considered SS group in this study was experiencing a lack of balance while performing the turning task.

**Angular velocity of flexion/extension** obtained by temporal processing of the sacrum, sternum and leading leg were 46% (*p* = 0.004), 61% (*p* = 0.001), 31% (*p* = 0.004) higher in the HI group, respectively (Table 3). However, the **range of motion** for upper body segments (sternum/sacrum) was larger in **flexion/extension and lateral bending**. Despite having a slower movement, this means SS covered a more extended angle as they performed the task. They might have difficulties maintaining upright posture and could show a lack of trunk control [15]. Slower upper body motion could be due to the neuromuscular deficiencies imposed by the stroke, such as muscle weakness, spasticity, and lack of coordination between different parts of the body [25]. Another reason for a more extensive range of motion in upper body segments of SS could be the lack of proper movement and motor control in affected body regions [26].

In agreement with the temporal processing, the **angular velocity in the flexion/extension** direction was more prominent in all the segments of HI after conducting two-partition spatial processing. However, the differences between the start, middle and end portions of the complete turning cycle can be observed (Table 4, Table 5 and Table 6). For example, the angular velocity of the trailing leg in flexion/extension was significantly lower in SS. However, the results of the four-partition analysis showed that it was similar at the end part of the task for both groups. In the case of the **range of motion in flexion/extension**, SS group showed higher values for sternum and sacrum in all instances of partitions. These results align with those of temporal processing, and the higher angles could be due to a ‘drag motion’ in the upper body to move the affected lower body regions of SS. There was no difference between the **range of motion** of leading/trailing legs in **flexion/extension** between SS and HI, except for the leading leg at the beginning of the task.

Meanwhile, higher angular velocity values **in rotation** were obtained for the HI, but in specific partitions during spatial processing. Both types of partitions, i.e., two and four, showed significantly different values in the first partition for the sternum sensor. Contrary to this, except for the last partition of the two and four-partition analyses, all partitions showed significantly higher values of **rotation angular velocity** (Table 4). This aligns with an earlier statement about HI being more stable, which may be the reason for the faster turning of the trunk in the first quarter of the turning cycle. However, both mid-section and upper body showed similar **rotation angular velocity** in both groups towards the end of the cycle, possibly meaning that both groups could decelerate and stop the core of their body at a similar rate. The lower body’s angular velocity in the rotation was larger in HI for the leading and trailing leg (except the last quarter of the four-partition). This could mean that the affected leg in SS and the trailing leg HI could decelerate similarly. Furthermore, despite having similar **angular velocity in lateral bending**, the **range of motion** in the same direction was almost double in SS vs. HI (Table 4 and Table 5). Finally, except for the last portion of the four-partition analysis of the complete turning cycle, this factor was significantly larger for the sacrum sensor in SS.

Apart from the kinematic measures of individual segments, their relative motion could provide in-depth insights into the synchronicity between the segments. This could be beneficial in determining the source of the movement discrepancies in cases where the motion of one segment is a combination of multiple segments connected in the form of a link. For example, movement variation in the sternum sensor could be due to issues in movement of the sacrum or relative motion in the sternum w.r.t sacrum. Analysis of both of these sensors (Table 4 and Table 5) showed that both segments displayed significant differences between the two groups in terms of **angular velocity and range of motion**. However, further analysis (Table 6) showed that the **relative angular velocity and range of motion** of sternum-sacrum were similar between the groups (except **angular velocity of rotation** in the first portion of all partitions). The differences in the first portion could be because the turning motion often starts with the torso rotation being the first movement during turning. Hence, excluding the first portion, we could conclude that source of the movement discrepancies between the stroke and the healthy group in the upper extremity could be the motion of the sacrum.

The general pattern of the measures in two-partition segmentation showed a decrease in angular velocity of the segments in all possible directions in the second half of turning (Table 4). This drop was more considerable in HI group, which could mean healthy individuals minimized the angular velocity of the segments towards the end of the task (>30% drop). To shed more light on this matter, the motion signals were segmented by 90°. Interestingly, the results of the four-partition segmentation demonstrated that in almost all joint and angular motions, there was an increase in angular velocity from part 1 to part 2 (Table 4). However, when the participants were halfway through the turning task, they began to decrease the angular velocity of the segments. This could mean that since, at the beginning, they did not know what the optimal motion would be, their neuromusculoskeletal system explored different patterns of muscle recruitments to figure out what would be the optimal pattern. When they reach the mid-point of the turn, they have enough information about optimal motion with less energy expenditure and motion, which led to a less angular velocity of the segments.

The movement data while participants performed a turning task was collected using IMU sensors, which are portable and offer out-of-the-lab evaluation benefits compared to the traditional camera-based systems. Moreover, these sensors are highly accessible and are often a part of several smart devices, such as smartphones and smartwatches that are now being used by most of the population. Thus, the presented work in this article can be extended towards determining the feasibility of utilizing smart devices instead of a specialized IMU-based system (used in this study) for detailed motion analysis of SS [27,28]. By enabling such analysis to be conducted out-of-the-lab using daily-used smart devices, highly accessible remote monitoring of recovery of SS can be possible. The outcomes of the investigations can then be used for developing/improving rehabilitation programs, and interventions/assistive devices, like an exoskeleton for effective gait recovery in SS. To this end, according to the highly affected measures of the SS group, the most critical part of neuromusculoskeletal system could be identified. These aspects could be taken into consideration in re/designing of the rehabilitation programs in the next phase of the project. To evaluate the efficiency of the new rehabilitation programs, our approach could also be implemented to screen the changes in the highly affected measures while the SS are going through the rehabilitation process. For example, in this study, the motion analysis results showed that the SS had less stability, showed by lower flexion–extension angular velocity of the four body segments and larger lateral bending range in sternum/sacrum compared to HI. Hence, there could be a rehabilitation program in which the focus will be enhancing the flexion–extension angular velocity of the upper extremity. Recording of the measures such as angular velocity and range of motion for sacrum and sternum before, while and after the rehabilitation period could help us to evaluate how the rehabilitation program is helpful to achieve the similar motion as HI in full turning. While this study implemented an IMU-based system for detecting movement changes in SS, similar signal processing techniques (temporal/spatial) can be implemented to detect other neuromusculoskeletal disorders.

Although this study provided insights about movement discrepancies in SS, the study is subject to a few limitations. In the findings of temporal processing of the range of motion of the leading leg in flexion/extension, similar values were obtained in the two groups. Even though this could mean that the movement of the leading leg was similar in the two groups, there can be three types of movement that can result into same values in the shank sensor. For instance, the human leg combines two segments (shank, thigh), and similar rotation angles can be obtained in the shank sensor in cases with movement of knee alone, movement of hip alone, and combination of hip and knee. Finally, the reasoning behind the movement discrepancies could be either due to neurological or musculoskeletal systems and pinpointing the exact issue from analyzing movement could be difficult. Nonetheless, the issue in the body region could be identified for further analysis, and/or, consideration in rehabilitation.

## 5. Conclusions

Understanding movement fluctuations in stroke survivors (SS) remains a crucial area for detecting the effects of affected neuromuscular systems. Among the activities of daily living, movement of on-spot turning, being among the most common, was studied in detail with the help of wearable sensors. The selected parameters in this study successfully differentiate between the movement performed by SS and show the level of progress/recovery as they are undergoing rehabilitation. The obtained data were segmented using both temporal and spatial processing methods. Due to the lack of observable patterns in the trailing leg signal, spatial processing can be used to differentiate between the movement of SS and healthy individuals. However, a number of partitions could play an important role while interpreting the results, and it is recommended to consider up to four partitions. This could enable differentiation between the start, mid, and end portions of the turning task. The motion analysis results show that the SS were less stable, demonstrated by lower flexion–extension angular velocity of the four body segments and more extensive lateral bending range of motion in sternum and sacrum compared to HI, as they performed the turning task. Meanwhile, findings of spatial processing showed similar movement as their healthy counterpart during the deceleration phase of the task. Moreover, a detailed analysis of relative movement between the sternum and sacrum showed that the sacrum’s source of the movement discrepancies. The outcomes of this study show that an in-depth analysis of a turning task can be conducted to study movement limitations in SS, and the findings of this study could help design targeted rehabilitation programs for SS.

## Figures and Tables

**Table 1:** Demographics of recruited participants.

Parameters	Gender		Age (SD) [year]	Height (SD) [cm]	Weight (SD) [kg]
Stroke Survivors (SS)	8 males	6 females	69 (8.4)	179 (8.3)	84.9 (22.3)
Healthy Individuals (HI)	6 males	8 females	74 (8.7)	162 (10)	68 (13)

**Table 2:** The selected parameters per segment in consideration for the analysis.

Segment	Parameters
Sternum and Sacrum	Angular Velocity in Flex/Ext Direction (°/sec)
	Angular Velocity in Lateral Bending Direction (°/sec)
	Angular Velocity in Rotation Direction (°/sec)
	Angular Velocity (Flex/Ext) of Sternum Relative to Sacrum (°/sec)
	Angular Velocity (Lateral Bending) of Sternum Relative to Sacrum (°/sec)
	Angular Velocity (Rotation) of Sternum Relative to Sacrum (°/sec)
	Range of Motion in Flex/Ext Direction (°)
	Range of Motion in Lateral Bending Direction (°)
	Range of Motion (Flex/Ext) for Sternum Relative to Sacrum (°)
	Range of Motion (Lateral Bending) for Sternum Relative to Sacrum (°)
	Range of Motion (Rotation) for Sternum Relative to Sacrum (°)
Shanks	Number of Cycles in the Turning Task
	Duration of the Turn (sec)
	Portion of Stance Phase in each Cycle (%)
	Angular Velocity in Flex/Ext Direction (°/sec)
	Angular Velocity in Rotation Direction (°/sec)
	Angular Velocity (Flex/Ext) of Leading/Trailing Leg (°/sec)
	Angular Velocity (Rotation) of Leading/Trailing Leg (°/sec)
	Range of Motion in Flex/Ext Direction
	Range of Motion (Flex/Ext) for Leading/Trailing Leg (°)

**Table 3:** Comparison between Stroke Survivors (SS) and Healthy Individual (HI) groups for the measures obtained from temporal processing of motion signals while turning. The significant measures’ *p*-values are shown in bold.

Parameters	SS (N = 14)	HI (N = 14)	*t*-Test (*p*-Value)
Number of Cycles	5.86 (1.56)	3.5 (0.94)	**0.001**
Duration of the Turn (sec)	7.67 (3.17)	3.72 (1.74)	**0.004**
Portion of Stance Phase in each Cycle (%)	53.28 (13.59)	38.96 (12.57)	**0.013**
Angular Velocity (Flex/Ext) of Sternum (°/sec)	0.89 (0.41)	1.43 (0.35)	**0.001**
Angular Velocity (Flex/Ext) of Sacrum (°/sec)	0.97 (0.44)	1.42 (0.34)	**0.004**
Angular Velocity (Flex/Ext) of Leading Leg (°/sec)	1.71 (0.37)	2.24 (0.37)	**0.004**
Range of Motion (Lateral Bending) for Sternum (°)	11.51 (5.03)	7.3 (3.48)	**0.021**
Range of Motion (Lateral Bending) for Sacrum (°)	8.88 (2.36)	7.31 (1.56)	**0.033**
Range of Motion (Flex/Ext) for Sternum (°)	8.69 (2.9)	5.24 (1.84)	**0.004**
Range of Motion (Flex/Ext) for Sacrum (°)	7.56 (2.73)	5.07 (1.28)	**0.014**
Range of Motion (Flex/Ext) for Leading Leg (°)	18.82 (7.64)	16.22 (5.73)	**0.324**

**Table 4:** Comparison between the angular velocity measures obtained from spatial processing of motion signals while turning among stroke survivors (SS) and healthy individuals (HI). The significant measures’ *p*-values are shown in bold. The dark pies depict the part of turning in consideration.

Parameters	Group	180° Division		Compare (%)	90° Division				Comparison between Partitions (%)		
		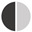	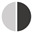	2/1	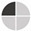	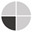	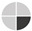	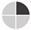	2/1	3/2	4/3
Angular Velocity (Flex/Ext) of Sternum (°/sec)	SS	0.77 (0.3)	0.67 (0.26)	−14	0.71 (0.32)	0.84 (0.32)	0.84 (0.3)	0.59 (0.28)	17	0	−35
	HI	1.85 (0.56)	1.08 (0.54)	−53	1.76 (0.480)	1.94 (0.67)	1.82 (0.61)	0.84 (0.56)	10	−6	−74
	*t*-test (*p*-value)	**<0.001**	**0.013**		**<0.001**	**<0.001**	**<0.001**	0.091			
Angular Velocity (Flex/Ext) of Sacrum (°/sec)	SS	0.82 (0.34)	0.72 (0.25)	−13	0.75 (0.33)	0.91 (0.37)	0.9 (0.32)	0.63 (0.25)	19	−1	−35
	HI	1.85 (0.54)	1.08 (0.51)	−53	1.77 (0.46)	1.94 (0.68)	1.84 (0.58)	0.83 (0.52)	9	−5	−76
	*t*-test (*p*-value)	**<0.001**	**0.022**		**<0.001**	**<0.001**	**<0.001**	0.155			
Angular Velocity (Flex/Ext) of Leading Leg (°/sec)	SS	0.8 (0.31)	0.7 (0.24)	−13	0.7 (0.32)	0.85 (0.42)	0.83 (0.32)	0.66 (0.25)	19	−2	-23
	HI	1.41 (0.58)	1.03 (0.47)	−31	0.38 (0.27)	1.8 (0.77)	1.42 (0.82)	0.82 (0.67)	130	−24	−54
	*t*-test (*p*-value)	**0.002**	**0.024**		**0.005**	**0.001**	**0.013**	0.369			
Angular Velocity (Flex/Ext) of Trailing Leg (°/sec)	SS	0.86 (0.36)	0.75 (0.23)	−14	0.83 (0.34)	1.08 (0.91)	1.01 (0.33)	0.64 (0.25)	26	−7	−45
	HI	2.26 (1.17)	1.13 (0.6)	−67	3.4 (1.06)	2.01 (1.67)	2.68 (2.19)	0.86 (0.47)	−51	29	−103
	*t*-test (*p*-value)	**0.001**	**0.026**		**<0.001**	0.111	**0.017**	0.109			
Angular Velocity (Rotation) of Sternum (°/sec)	SS	0.23 (0.22)	0.18 (0.15)	−24	0.21 (0.18)	0.26 (0.25)	0.26 (0.23)	0.15 (0.12)	21	0	−54
	HI	0.4 (0.24)	0.21 (0.1)	−62	0.37 (0.23)	0.42 (0.26)	0.39 (0.21)	0.15 (0.1)	13	−7	−89
	*t*-test (*p*-value)	**0.047**	0.626		**0.028**	0.081	0.064	0.899			
Angular Velocity (Rotation) of Sacrum (°/sec)	SS	0.15 (0.1)	0.14 (0.09)	−7	0.14 (0.09)	0.17 (0.13)	0.17 (0.1)	0.13 (0.1)	19	0	−27
	HI	0.39 (0.26)	0.24 (0.22)	−48	0.36 (0.23)	0.4 (0.28)	0.4 (0.31)	0.19 (0.21)	11	0	−71
	*t*-test (*p*-value)	**0.009**	0.107		**0.007**	**0.01**	**0.018**	0.232			
Angular Velocity (Rotation) of Leading Leg (°/sec)	SS	0.06 (0.05)	0.05 (0.07)	−18	0.05 (0.04)	0.06 (0.06)	0.05 (0.07)	0.06 (0.07)	18	−18	18
	HI	0.23 (0.17)	0.17 (0.12)	−30	0.13 (0.09)	0.3 (0.21)	0.21 (0.17)	0.16 (0.14)	79	−35	−27
	*t*-test (*p*-value)	**0.004**	**0.001**		**0.01**	**0.001**	**0.005**	**0.012**			
Angular Velocity (Rotation) of Trailing Leg (°/sec)	SS	0.06 (0.04)	0.04 (0.04)	−40	0.08 (0.07)	0.12 (0.16)	0.05 (0.04)	0.07 (0.05)	40	−82	33
	HI	0.34 (0.24)	0.1 (0.06)	−109	0.48 (0.3)	0.28 (0.23)	0.44 (0.48)	0.11 (0.16)	−53	44	−120
	*t*-test (*p*-value)	**0.001**	**0.004**		**<0.001**	0.056	**0.011**	0.443			
Angular Velocity (Lateral Bending) for Sternum (°)	SS	0.13 (0.14)	0.09 (0.07)	−36	0.13 (0.16)	0.13 (0.11)	0.13 (0.13)	0.09 (0.09)	0	0	−36
	HI	0.15 (0.09)	0.07 (0.05)	−73	0.14 (0.14)	0.16 (0.1)	0.13 (0.08)	0.06 (0.05)	13	−21	−74
	*t*-test (*p*-value)	0.619	0.428		0.879	0.419	0.976	0.297			
Angular Velocity (Lateral Bending) for Sacrum (°)	SS	0.05 (0.03)	0.04 (0.03)	−22	0.04 (0.03)	0.07 (0.1)	0.06 (0.03)	0.04 (0.02)	55	−15	−40
	HI	0.14 (0.14)	0.09 (0.08)	−43	0.17 (0.15)	0.13 (0.13)	0.14 (0.13)	0.08 (0.08)	−27	7	−55
	*t*-test (*p*-value)	**0.037**	0.101		**0.012**	0.258	**0.049**	0.13			

**Table 5:** Comparison between the range of motion measures obtained from spatial processing of motion signals while turning among stroke survivors (SS) and healthy individuals (HI). The significant measures’ *p*-values are shown in bold. The dark pies depict the part of turning in consideration.

Parameters	Group	180° Division		Compare (%)	90° Division				Comparison between Partitions (%)		
		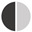	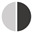	2/1	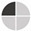	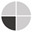	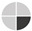	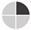	2/1	3/2	4/3
Range of Motion (Flex/Ext) for Sternum (°)	SS	7.07 (2.76)	7.77 (2.93)	9	6.03 (2.96)	5.97 (2.67)	5.55 (2.47)	6.77 (2.82)	−1	−7	20
	HI	4.41 (1.58)	4.42 (1.68)	0	1.84 (0.94)	3.6 (1.9)	3.15 (1.82)	3.65 (1.32)	65	−13	15
	*t*-test (*p*-value)	**0.007**	**0.006**		**< 0.001**	**0.01**	**0.03**	**0.002**			
Range of Motion (Flex/Ext) for Sacrum (°)	SS	6.18 (2.71)	6.88 (2.57)	11	5.28 (2.49)	5.14 (2.63)	5.48 (2.07)	6.13 (2.57)	−3	6	11
	HI	3.41 (1.3)	4.13 (1.43)	19	1.85 (1.03)	3.08 (1.42)	2.92 (1.47)	3.65 (1.54)	50	−5	22
	*t*-test (*p*-value)	**0.005**	**0.005**		**<0.001**	**0.039**	**0.008**	**0.005**			
Range of Motion (Flex/Ext) for Leading Leg (°)	SS	17.11 (7.77)	17.84 (8.37)	4	13.8 (6.23)	14.73 (8.99)	15.67 (8.24)	15.78 (7.74)	7	6	1
	HI	10.64 (5.59)	14.98 (6.31)	34	3.25 (2.87)	9.85 (6.49)	9.62 (7.92)	12.83 (3.74)	101	−2	29
	*t*-test (*p*-value)	**0.031**	0.342		**<0.001**	0.163	0.08	0.214			
Range of Motion (Flex/Ext) for Trailing Leg (°)	SS	19.11 (6.49)	20.19 (6.51)	5	18.04 (7.15)	16.77 (6.22)	17.68 (7.7)	17.76 (6.01)	−7	5	0
	HI	23.1 (11.45)	23.36 (10.14)	1	16.6 (7.64)	20.23 (11.52)	19.24 (9.74)	19.33 (7.75)	20	−5	0
	*t*-test (*p*-value)	0.244	0.272		0.633	0.305	0.571	0.555			
Range of Motion (Lateral Bending) for Sacrum (°)	SS	7.54 (2.85)	7.65 (2.49)	1	6.02 (2.7)	6.04 (1.96)	5.63 (1.51)	7.15 (2.76)	0	−7	24
	HI	5.47 (1.63)	6.71 (2.66)	20	3.03 (1.2)	4.46 (1.87)	3.62 (2.06)	5.81 (3.21)	38	−21	46
	*t*-test (*p*-value)	**0.002**	0.304		**0.002**	**0.012**	**0.003**	0.21			
Range of Motion (Lateral Bending) for Sternum (°)	SS	9.46 (5.81)	12.82 (7.76)	30	7.65 (5.47)	7.47 (5.98)	6.72 (4.38)	12.33 (8.02)	−2	−11	59
	HI	4.65 (2.01)	6.89 (3.69)	39	2.37 (1.78)	4.09 (1.99)	3.58 (1.87)	5.88 (3.82)	53	−13	49
	*t*-test (*p*-value)	**0.005**	**0.02**		**0.006**	**0.028**	**0.04**	**0.012**			

**Table 6:** Comparison between the relative range of motion and angular velocity measures between sensors obtained from spatial processing of motion signals while turning among stroke survivors (SS) and healthy individuals (HI). The significant measures’ *p*-values are shown in bold. The dark pies depict the part of turning in consideration.

Parameters	Group	180° Division		Compare (%)	90° Division				Comparison between Partitions (%)		
		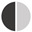	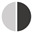	2/1	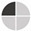	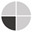	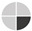	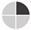	2/1	3/2	4/3
Angular Velocity (Flex/Ext) of Leading/Trailing Leg (°/sec)	SS	0.95 (0.13)	0.94 (0.16)	−1	0.85 (0.22)	1.03 (0.36)	0.8 (0.15)	1.05 (0.28)	19	−25	27
	HI	0.74 (0.3)	0.96 (0.19)	26	0.17 (0.27)	1.22 (0.54)	0.95 (0.6)	0.95 (0.51)	151	−25	0
	*t*-test (*p*-value)	**0.016**	0.692		**<0.001**	0.295	0.352	0.558			
Angular Velocity (Rotation) of Leading/Trailing Leg (°/sec)	SS	5.32 (14)	18.52 (55.66)	111	4.72 (8.8)	2.21 (4.21)	4.16 (6.08)	1.58 (2.32)	−72	61	−90
	HI	1.05 (1.51)	2.01 (1.65)	63	0.32 (0.2)	1.92 (3.09)	10.71 (35.84)	12.25 (32.22)	143	139	13
	*t*-test (*p*-value)	0.28	0.289		0.084	0.838	0.521	0.24			
Range of Motion (Flex/Ext) for Leading/Trailing Leg (°)	SS	1.11 (0.87)	1.07 (0.78)	−4	1.04 (0.96)	1.15 (1.04)	1.24 (1.1)	1.18 (1.07)	10	8	−5
	HI	0.87 (0.99)	0.93 (0.89)	7	0.34 (0.56)	1.08 (1.55)	1.23 (1.97)	0.87 (0.68)	104	13	−34
	*t*-test (*p*-value)	0.544	0.671		**0.037**	0.91	0.993	0.323			
Range of Motion (Rotation) for Sternum Relative to Sacrum (°)	SS	21.94 (20.77)	21.27 (14.21)	−3	46.09 (42.95)	25.24 (25.23)	33.81 (56.7)	28.25 (19.97)	−58	29	−18
	HI	24.28 (61.91)	9.9 (20.09)	−84	39.3 (65)	21.85 (62.21)	6.45 (4.6)	13.97 (31.75)	−57	−109	74
	*t*-test (*p*-value)	0.898	0.134		0.752	0.861	0.088	0.216			
Range of Motion (Lateral Bending) for Sternum Relative to Sacrum (°)	SS	14.64 (11.72)	15.84 (11.97)	8	14.68 (11.25)	14.74 (11.96)	15.83 (12.46)	15.97 (11.9)	0	7	1
	HI	12.26 (10.4)	11.98 (10.24)	−2	12.4 (10.61)	12.34 (10.36)	12.81 (10.69)	11.83 (10.09)	0	4	−8
	*t*-test (*p*-value)	0.576	0.385		0.59	0.574	0.495	0.349			
Range of Motion (Flex/Ext) for Sternum Relative to Sacrum (°)	SS	9.05 (7.34)	8.77 (7.27)	−3	9.04 (7.33)	9.02 (7.32)	8.69 (7.13)	8.81 (7.41)	0	−4	1
	HI	5.75 (4.33)	5.2 (4.14)	−10	5.88 (4.39)	5.82 (4.31)	5.05 (3.52)	5.28 (4.13)	−1	−14	4
	*t*-test (*p*-value)	0.095	0.111		0.086	0.114	0.096	0.122			
Angular Velocity (Rotation) of Sternum Relative to Sacrum (°/sec)	SS	0.19 (0.16)	0.18 (0.14)	−5	0.18 (0.15)	0.21 (0.17)	0.24 (0.18)	0.16 (0.12)	15	13	−40
	HI	0.44 (0.44)	0.26 (0.33)	−51	0.41 (0.38)	0.45 (0.47)	0.44 (0.44)	0.2 (0.31)	9	−2	−75
	*t*-test (*p*-value)	**0.048**	0.417		**0.038**	**0.037**	0.08	0.598			
Angular Velocity (Lateral Bending) of Sternum Relative to Sacrum (°/sec)	SS	0.14 (0.15)	0.1 (0.07)	−33	0.13 (0.15)	0.17 (0.17)	0.15 (0.14)	0.09 (0.07)	27	−13	−50
	HI	0.17 (0.15)	0.11 (0.08)	−43	0.18 (0.19)	0.17 (0.14)	0.19 (0.13)	0.09 (0.07)	−6	11	−71
	*t*-test (*p*-value)	0.613	0.848		0.398	0.947	0.343	0.938			
Angular Velocity (Flex/Ext) of Sternum Relative to Sacrum (°/sec)	SS	0.07 (0.07)	0.06 (0.06)	−15	0.05 (0.05)	0.09 (0.11)	0.08 (0.09)	0.05 (0.05)	57	−12	−46
	HI	0.06 (0.06)	0.03 (0.03)	−67	0.06 (0.05)	0.09 (0.09)	0.05 (0.04)	0.03 (0.03)	40	−57	−50
	*t*-test (*p*-value)	0.866	0.186		0.296	0.844	0.327	0.274			

## Data Availability

All data included in this study are available upon request by contact with the corresponding author.
